# A Novel Strategy for Creating an Antibacterial Surface Using a Highly Efficient Electrospray-Based Method for Silica Deposition

**DOI:** 10.3390/ijms23010513

**Published:** 2022-01-03

**Authors:** Odelia Levana, Soonkook Hong, Se Hyun Kim, Ji Hoon Jeong, Sung Sik Hur, Jin Woo Lee, Kye-Si Kwon, Yongsung Hwang

**Affiliations:** 1 Soonchunhyang Institute of Medi-Bio Science (SIMS), Soonchunhyang University, Cheonan-si 31151, Chungnam-do, Korea; odelialevana@sch.ac.kr (O.L.); jjh2020@sch.ac.kr (J.H.J.); sstahur@sch.ac.kr (S.S.H.); 2 Department of Integrated Biomedical Science, Soonchunhyang University, Asan-si 31538, Chungnam-do, Korea; 3 Department of Mechanical and Naval Architectural Engineering, Republic of Korea Naval Academy, Changwon-si 51704, Kyungsangnam-do, Korea; hsk753@mnd.go.kr; 4 Department of Electronic Materials, Devices and Equipment Engineering, Soonchunhyang University, Asan-si 31538, Chungnam-do, Korea; ksy9046@naver.com; 5 Department of Molecular Medicine, Gachon University College of Medicine, Incheon 21936, Korea; jwlee@gachon.ac.kr; 6 Department of Mechanical Engineering, Soonchunhyang University, Asan-si 31538, Chungnam-do, Korea

**Keywords:** electrospray, antibacterial surface, hydrophobicity, silica deposition, anti-adhesive properties

## Abstract

Adhesion of bacteria on biomedical implant surfaces is a prerequisite for biofilm formation, which may increase the chances of infection and chronic inflammation. In this study, we employed a novel electrospray-based technique to develop an antibacterial surface by efficiently depositing silica homogeneously onto polyethylene terephthalate (PET) film to achieve hydrophobic and anti-adhesive properties. We evaluated its potential application in inhibiting bacterial adhesion using both Gram-negative *Escherichia coli* (*E. coli*) and Gram-positive *Staphylococcus aureus* (*S. aureus*) bacteria. These silica-deposited PET surfaces could provide hydrophobic surfaces with a water contact angle greater than 120° as well as increased surface roughness (root mean square roughness value of 82.50 ± 16.22 nm and average roughness value of 65.15 ± 15.26 nm) that could significantly reduce bacterial adhesion by approximately 66.30% and 64.09% for *E. coli* and *S. aureus*, respectively, compared with those on plain PET surfaces. Furthermore, we observed that silica-deposited PET surfaces showed no detrimental effects on cell viability in human dermal fibroblasts, as confirmed by MTT (3-(4,5-dimethylthiazol-2-yl)-2,5 diphenyl tetrazolium bromide and live/dead assays. Taken together, such approaches that are easy to synthesize, cost effective, and efficient, and could provide innovative strategies for preventing bacterial adhesion on biomedical implant surfaces in the clinical setting.

## 1. Introduction

Biofilms are sessile bacterial aggregates that firmly adhere to both living and nonliving surfaces [1]. Bacterial adhesion is modulated by physical forces, including non-specific van der Waals, Lewis acid–base, and electrostatic forces, or bacterial appendages, such as pili or flagella [2,3]. Newly formed biofilms at the interfaces of a material surface or living body produce an exopolysaccharide (EPS) matrix, which accelerates the continuous growth, maturation, and propagation from the original region to other locations by strengthening cell–cell, cell–tissue, and cell–surface interactions as well as increases stress resistance against harsh conditions, such as heat and acidic shock [4]. Moreover, the EPS matrix consists of polymeric substances that can retard antibiotic penetration, which eventually increases antimicrobial resistance and causes difficulties in clinical treatment [5,6]. Therefore, a preventive strategy is required to inhibit initial bacterial attachment and their continuous growth.

Bactericidal agents, such as antibiotics and biocides, can be effectively applied on biocidal surfaces; however, the accumulation of dead bacteria and other debris on the surface could lead to the initiation of acute inflammation [7,8]. To overcome this drawback, the incorporation of metal chelators, such as ethylenediaminetetraacetic acid and citrate, onto the surface has been reported to disrupt bacterial surface adherence without killing the cells directly [9,10]. Another study by Asadi et al. [11] demonstrated the development of sugar-based host receptor analogs with the potential to weaken bacterial adhesion to the surface. Among these approaches, the fabrication of hydrophobic surfaces with anti-adhesive properties has been extensively studied, particularly in the healthcare industry, because bacterial infections are often caused by medical equipment [12].

Bacterial adhesion on the surface is mediated by surface wettability, and various methods, such as lithography, etching, templating, sol-gel method, layer-by-layer deposition, and spray coating, have been used to generate hydrophobic surfaces [13,14,15]. Among these techniques, electrospray has been widely used to create hydrophobic surfaces with antibacterial properties [16]. Nanoparticles are commonly utilized for electrospray-based coatings; for example, titania (TiO_2_) effectively inhibits *Escherichia coli* (*E. coli*) and *Staphylococcus aureus* (*S. aureus*) adhesion on surfaces owing to the hydrophobic properties of the nanoparticles, as reported in previous studies [16,17]. Moreover, silica (SiO_2_) shows good biocompatibility and has been developed as coating materials, particularly in biomedical applications, such as urinary catheters and dental implants [18,19,20].

Polyethylene terephthalate (PET) has been widely used in the healthcare industry due to its mechanical properties, such as high biocompatibility and uniformity, as well as high mechanical strength [21]. However, bacterial contamination on PET surface is one of the drawbacks and has been commonly reported from its application in medical devices [22]. Therefore, in this study, we aimed to develop a novel electrospray technique to create a hydrophobic silica-rich surface with anti-adhesive properties and evaluate its potential as an antibacterial surface to inhibit initial bacterial adhesion. PET substrate was deposited with silica using a highly efficient electrospray-based method, and sample characterization was performed using scanning electron microscopy (SEM), Fourier-transform infrared spectroscopy (FTIR), and atomic force microscopy (AFM), whereas hydrophobicity was analyzed by water contact angle (WCA) measurements. The antibacterial properties of the fabricated surface were investigated using *E. coli* and *S. aureus* as Gram-negative and Gram-positive model bacteria, respectively. Our method effectively inhibited bacterial adhesion on the surface without any cytotoxic effects on human dermal fibroblasts, as assessed by cytotoxicity tests. Therefore, our findings could be applied in biomedical applications as an alternative method of antibiotic usage.

## 2. Results

### 2.1. Fabrication of a SiO_2_-Deposited Surface Using an Electrospray Technique

**Figure 1:**
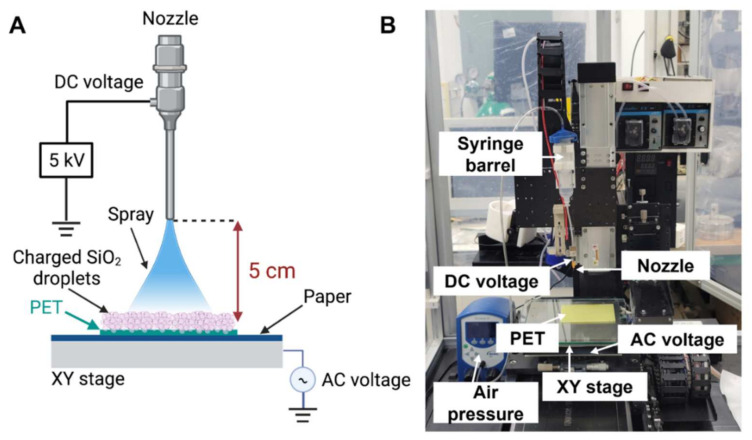
Fabrication and characterization of the electrosprayed SiO_2_ surface. (**A**) A schematic illustration of electrospray SiO_2_-deposited surface fabrication on polyethylene terephthalate (PET) substrate (created with BioRender.com). (**B**) A photograph of the experimental set-up.

In this study, we focused on the fabrication of silica-deposited PET using the novel alternating current (AC)-biased method for efficiently creating homogenous hydrophobic surfaces, as displayed in Figure 1. Silica (SiO_2_) solution was prepared as the material for electrospray coating and loaded into a nozzle. A low air pressure with 2.0 kPa and high DC voltage (5 kV) was applied to the syringe barrel in order to prevent the dripping of large droplets from the nozzle, resulting in sprayed charge droplets that were easily attached to PET surfaces. In contrast to the conventional electrospray method, we applied AC voltage under the substrate holder as a replacement of ground (zero) voltage to improve the uniformity of electrospray-based silica deposition and reduce the effect of undissipated charges.

### 2.2. Characterization of the SiO_2_-Deposited Surface by SEM, FTIR, and AFM

**Figure 2:**
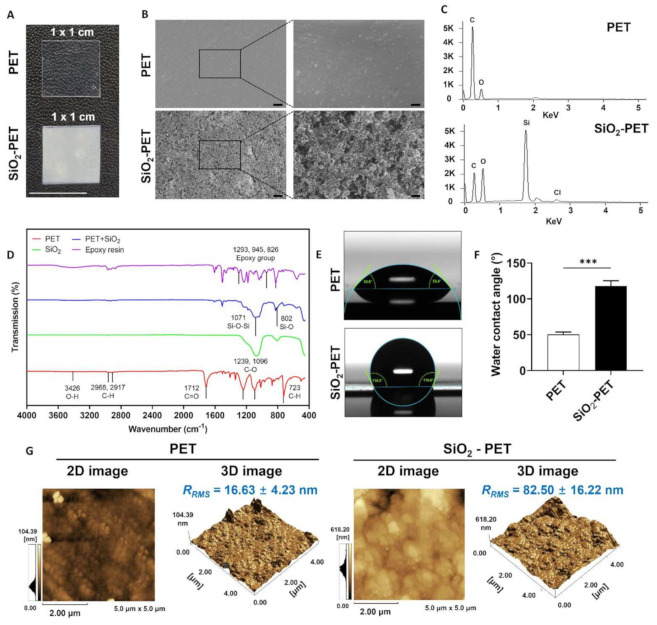
Characterization of the SiO_2_-electrosprayed surface. (**A**) Optical images of plain PET and SiO_2_-deposited PET films (scale bar: 10 mm). (**B**) SEM images of plain PET and SiO_2_-deposited PET films (scale bar: 2 μm (left) and 400 nm (right)). (**C**) EDS analysis of PET and SiO_2_-deposited PET. (**D**) FTIR analysis PET and SiO_2_-deposited PET. (**E**) Water droplets formed on PET and SiO_2_-deposited PET. (**F**) Water contact angles of PET and SiO_2_-deposited PET surfaces. (**G**) AFM images of plain PET and SiO_2_-deposited PET films roughness (*n* = 5), *** *p* < 0.001.

As reported in our previous study, the novel AC-biased electrospray technique could result in high uniformity and homogenous deposition of silica on various substrates including PET, glass, and paper with hydrophobic surface properties [23]. We further confirmed these findings by SEM and FTIR to validate the effectiveness of our novel coating method. SEM analysis was carried out to investigate the morphology of the SiO_2_-deposited PET in comparison with that of the plain PET surface. As shown in Figure 2A, the SiO_2_-deposited PET surface was completely covered by granular and pebbled structures of SiO_2_ particles without any empty space. In contrast, a smooth topology and no SiO_2_ particles were observed on the plain PET surface. Additionally, as shown in Supplementary Figure S1 (see Supplementary Materials), cross-sectional SEM images displayed 1.71 µm thickness of SiO_2_ layer on PET substrate. In contrast with the deposited surface, plain PET exhibits no deposition of SiO_2_, suggesting that the deposition of SiO_2_ onto PET substrate was successful. We further conducted energy dispersive spectroscopy analysis to assess the chemical compositions of plain PET and SiO_2_-deposited PET surfaces. The Si and Cl atoms were detected only on SiO_2_-deposited PET because silica and epoxy resin solutions were used as the deposition materials (Figure 2B).

In addition, FTIR analysis was used to determine the functional groups presented on the fabricated surface. A representative spectrogram is shown in Figure 2C and Supplementary Figure S2. To confirm the effectiveness of the electrospray deposition onto PET films, we characterized the SiO_2_ powder and compared it with the fabricated surface. Because SiO_2_ powder was initially mixed with epoxy resin to increase its adhesion on PET surface, the bands at 1293, 945, and 826 cm^−1^ were shown as characteristic bands for epoxy groups, and bands were presented in SiO_2_-deposited PET and epoxy resin but not in plain PET surface [24]. Absorption bands at 1071 and 802 cm^−1^ were presented in the SiO_2_ powder and on the SiO_2_-deposited PET and were attributed to Si-O-Si and Si-O groups, respectively [25,26,27]. Moreover, the FTIR spectrum of the plain PET surface showed multiple bands of C=O and C-H groups at 1712 and 723 cm^−1^ [28], respectively, as well as C-O groups at 1239 and 1096 cm^−1^ [29,30]. Furthermore, Si-O-Si and Si-O bands did not appear in the plain PET samples. However, absorption bands at 723, 1096, 1239, and 1712 cm^−1^ decreased or disappeared because of the electrospray coating.

Substrate wettability is the primary factor that influences bacterial adhesion; hydrophobic surfaces show reduced bacterial attachment, whereas hydrophilic surfaces promote bacterial adhesion [14,15]. In this study, we conducted WCA measurements to evaluate the hydrophobicity of the SiO_2_-deposited PET surface as compared with that of the plain PET surface. The WCA was calculated at room temperature (20 °C) using an optical contact angle meter. As shown in Figure 2D–F, the water droplet on the plain PET surface was spread, whereas the water droplet on the SiO_2_-deposited PET was rounded and aggregated. SiO_2_ deposition on the PET surface exhibited a decrease in water wettability with a higher WCA value (118.1° ± 7.2°) compared with the plain PET sample (50.3° ± 3.5°) (Supplementary Figure S3). We also performed a dynamic contact angle test to evaluate the degree of stickiness of both plain PET and SiO_2_-deposited PET surfaces. Additionally, higher water repellency was observed on the SiO_2_-deposited PET sample as water droplets easily rolled off from the surface at 45° inclination. Meanwhile, the plain PET sample with lower WCA value allowed the spreading of water droplets and slow movement even on the tilted surface (Supplementary Information Video S1 and S2). These findings indicate that surface hydrophobicity was achieved by the electrosprayed SiO_2_ deposition.

As deposition of coating materials has been reported to affect surface topography [31,32,33,34], we performed AFM to analyze the roughness of our fabricated surface. As shown in Figure 2G, on one hand, the deposition of SiO_2_ onto PET substrate showed root-mean-square roughness (*R_RMS_*) and average roughness (*R_a_*) values of 82.50 ± 16.22 nm and 65.15 ± 15.26 nm, respectively. Conversely, the plain PET sample showed *R_RMS_* = 16.63 ± 4.23 nm and *R_a_* = 11.65 ± 2.87, which were significantly lower than those of the SiO_2_-deposited PET sample. These results implied that the deposition of SiO_2_ onto PET substrate increased surface roughness as well as hydrophobicity, which might be able to synergistically contribute to antibacterial properties.

### 2.3. Antibacterial Properties of SiO_2_-Deposited Surfaces

**Figure 3:**
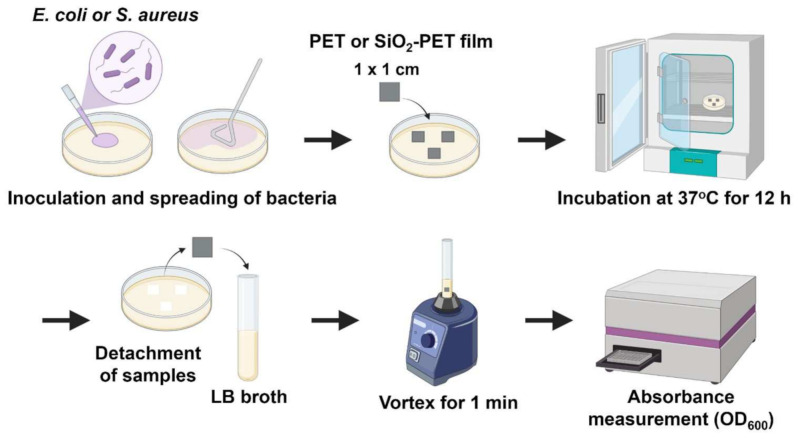
A schematic illustration of the antibacterial assay—created with BioRender.com (accessed on 25 October 2021).

Here, we further hypothesized that the hydrophobicity of silica may prevent bacterial adhesion to the surface. To determine the antibacterial properties of our hydrophobic fabricated surface, we performed antibacterial assays using *E. coli* and *S. aureus* as model bacteria. As shown in Figure 3, both *E. coli* and *S. aureus* were initially cultured on Luria-Bertani (LB) agar, then plain PET and SiO_2_-deposited PET samples sized 1 × 1 cm were directly placed on top of the agar to allow bacterial adhesion on the surfaces. The amount of adhered bacteria were measured from the surfaces of both plain PET and SiO_2_-deposited PET films; after transferring the films to LB broth tubes and applying vortex, the detached bacteria from the surfaces were measured, whereas both *E. coli* and *S. aureus* grown on the surface of agar were used as positive controls (Figure 3).

**Figure 4:**
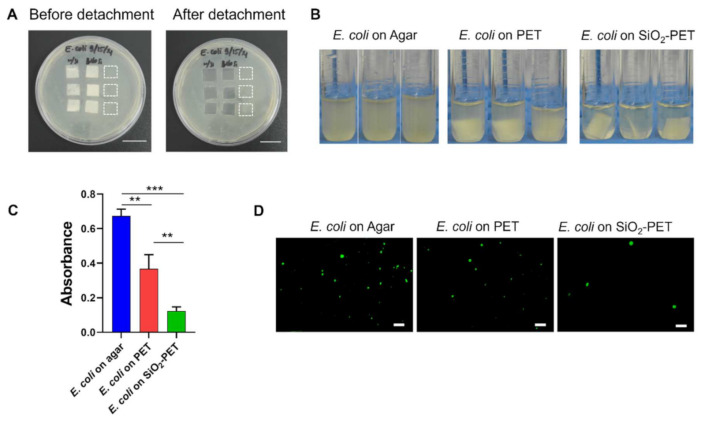
Assessment of the antibacterial properties of electrosprayed SiO_2_ films against *E. coli*. (**A**) Images of agar plates before and after film detachment (scale bar: 20 mm). (**B**) Images of detached films and agar in LB broth after vortexing for 1 min. (**C**) Quantitative analysis of the detached films based on absorbance at 600 nm (OD_600_). ** *p* < 0.01, *** *p* < 0.001. (**D**) Fluorescence images of live *E. coli* on the surface (scale bar: 200 µm).

**Figure 5:**
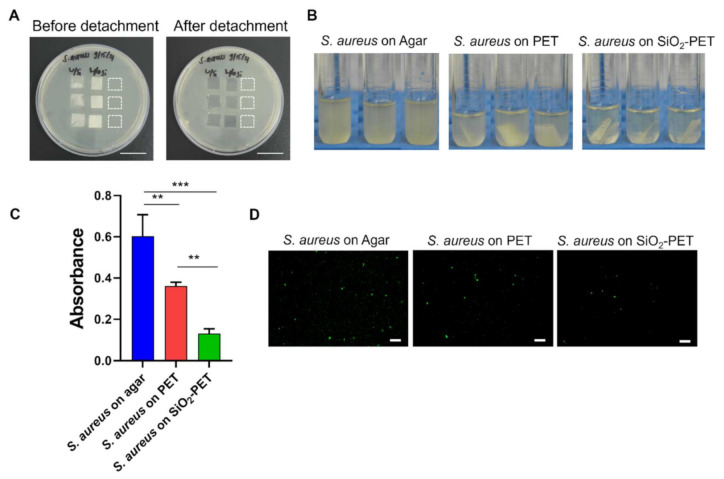
Assessment of the antibacterial properties of electrosprayed SiO_2_ films against *S. aureus*. (**A**) Images of agar plates before and after film detachment (scale bar: 20 mm). (**B**) Images of detached films and agar in LB broth after vortexing for 1 min. (**C**) Quantitative analysis of detached films based on absorbance at 600 nm (OD_600_). ** *p* < 0.01, *** *p* < 0.001. (**D**) Fluorescence images of live *S. aureus* on the surface (scale bar: 200 µm).

As shown in Figure 4A and Figure 5A, both *E. coli* and *S. aureus* were able to grow rapidly on the surface of LB agar that was not covered by films for 12 h of incubation. After detaching adhered bacteria (*E. coli* and *S. aureus*) on to the samples, the SiO_2_-deposited PET suspension had the lowest turbidity among all groups, whereas the bacterial suspension from the PET sample was more opaque than that of the SiO_2_-deposited PET. Moreover, the agar-grown *E. coli* and *S. aureus* (positive control) suspensions appeared to be turbid compared to the other groups (Figure 4B and Figure 5B). This qualitative observation was further confirmed by an absorbance measurement, which is shown in Figure 4C and Figure 5D. Compared to the plain PET and SiO_2_-deposited PET samples, agar-grown bacteria (positive control) displayed the highest absorbance with OD_600_ values of 0.67 A and 0.60 A for *E. coli* and *S. aureus*, respectively. The OD_600_ value of adhered *E. coli* on to SiO_2_-deposited PET was 0.12, whereas plain PET sample showed higher bacterial adhesion (0.37) (Figure 4C). The same trend was also evidenced for *S. aureus* as SiO_2_-deposited PET showed the lowest OD_600_ value of 0.13 compared to plain PET samples (0.36) (Figure 5C). Based on these absorbance results, the inhibition percentages of SiO_2_-deposited PET against both bacteria were quite similar, with inhibition percentages of 66.30% and 64.09% for *E. coli* and *S. aureus*, respectively, suggesting that our fabricated surfaces are effective in inhibiting both Gram-positive and Gram-negative bacterial adhesion.

Additionally, because bacterial aggregation on the surface is a precursor of biofilm formation [1], we conducted fluorescence imaging for both *E. coli* and *S. aureus* to observe a reduction in bacterial aggregates and their spatial distribution onto the plain and SiO_2_-deposited PET samples. The visualization of *E. coli* on the surface was performed without any staining procedure as we used the GFP-tagged *E. coli* strain. Consistent with the quantitative absorbance results, on the one hand, the *E. coli* grown on agar in the same area as the samples (positive control) showed the highest number of viable cells (Figure 4D). Conversely, SiO_2_-deposited PET demonstrated a significant reduction of *E. coli* attachment, while plain PET surface allowed bacterial attachment after 12 h of culture. For the visualization of live *S. aureus*, we performed live/dead staining and similar to the *E. coli* results, significantly increased numbers of *S. aureus* colonies were observed in the positive control, whereas SiO_2_-deposited PET showed the least adhesion among all the groups (Figure 5D). Together, these results implied that the electrospray deposition of SiO_2_ onto PET surface could limit bacterial adhesion.

### 2.4. Cytotoxicity Test for In Vitro Cell Culture

**Figure 6:**
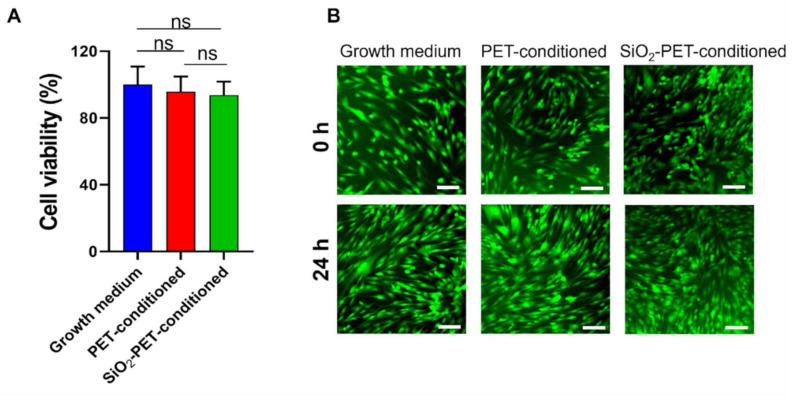
Cytotoxicity tests for PET and SiO_2_-deposited PET using human dermal fibroblasts (HDFs). (**A**) Cell viability after 24 h growth. (**B**) Fluorescence images of live/dead staining of HDFs at passage after 0 h and 24 h of incubation in growth medium or conditioned medium (scale bar: 100 µm). “ns” indicates that there is no statistical difference.

To assess whether deposited SiO_2_ has a detrimental effect on cell viability, we conducted an MTT assay and analyzed cell viability after incubating human dermal fibroblasts for 24 h in media preconditioned with plain PET and SiO_2_-deposited PET samples as compared with growth medium (Figure 6). In this study, we evaluated the cytotoxicity of plain PET and SiO_2_-deposited PET samples against human dermal fibroblasts (HDFs) because skin is the body’s largest external organ and a host organ for diverse microorganisms, including bacteria, and some of them, such as *S. aureus* and *Streptococcus pyogenes*, are pathogens that can cause infections [35,36]. As shown in Figure 6, there were no significant changes in HDF viability after 24 h of exposure in PET and SiO_2_-deposited PET conditioned media, with cells showing 95.87% and 93.75% viability, respectively. 

In addition to the MTT assay, these findings were further corroborated by live/dead staining results. As shown in Figure 6B, live HDF cells were visualized by green fluorescence and cell morphology showed no signs of cell death and morphological changes due to any substances released from the PET and SiO_2_-deposited PET films. Moreover, the cells were able to grow rapidly in the presence of the conditioned medium for 24 h. Therefore, these qualitative and quantitative data are evidence for the highly biocompatible properties of SiO_2_-electrosprayed surface.

## 3. Discussion

Silica (SiO_2_) is non-toxic, highly flexible, and chemically stable and exhibits good biocompatibility and bio-inert properties [37]. In addition to biocompatibility, silica also displays bioconjugation properties, and silica nanoparticles are commonly introduced as coating materials [38]. Thus, various approaches have been used in biomedical, diagnostic, and therapeutic applications, particularly for the coating of various medical devices [18,19,39]. Furthermore, Wang et al. [19] developed a silica-based antibacterial coating for dental implants and showed that silica nanoparticles and gentamycin could be successfully incorporated without any risk of infection, demonstrating that silica-based materials are applicable as a drug delivery system.

The fabrication of hydrophobic surfaces has been developed using numerous techniques, such as lithography, etching, templating, sol-gel method, layer-by-layer deposition, and spray coating [13]. Among these options, electrospray-based coating techniques have been widely used for coating thin films with nanomaterials by applying a high voltage known as the electrospray technique, resulting in charged droplet production from the aerosol phase [16,39]. This method has the advantage of creating a highly uniform coating on a wide variety of surfaces through an interplay between the nanoparticle deterministic velocity and thermal velocity due to Brownian motion [40]. Furthermore, the electrospray technique can be easily scaled up for industrial processes [41]. In contrast to the conventional electrospray method, we successfully developed a novel technique for generating SiO_2_-deposited surfaces by applying an alternating current (AC) voltage under the substrate holder (Figure 1). According to our preliminary studies, the effectiveness of this method was demonstrated by an increase in the coating layer thickness to 1.91 μm, implying that the AC voltage yielded a high uniformity and deposition rate. Furthermore, substrates with a coating thickness of 1 µm exhibit hydrophobic properties and are capable of ensuring reliability in harsh environments [23]. In the same study, we also assessed the stability and durability of SiO_2_-deposited surfaces by sandpaper abrasion, tape peeling, and UV irradiation tests as well as immersion in a corrosive liquid. The stability of SiO_2_-deposited samples was shown by no significant changes of water contact angle values upon UV light exposure for 24 h and after tape peeling and multiple abrasion cycles. Meanwhile, the durability test was performed by immersing the samples in hydrochloric acid (HCl) for 20 min. Although the water contact angle values gradually decrease in an immersion time-dependent manner, we observed only a slight (less than 3%) decrease in WCA when samples were exposed for 10 min [23].

Previously, various types of superhydrophobic surfaces, such as window glass and solar cell panels, were developed for self-cleaning applications based on their ability to weaken bacterial adhesion [42]. The wettability of a surface is determined by the WCA value; a surface is considered hydrophobic if the WCA is in the range of 90–150° and is defined as superhydrophobic when the WCA is greater than 150° [43]. Although silicon itself is not inherently antibacterial, SiO_2_-deposited surfaces can effectively inhibit bacterial adhesion owing to their hydrophobic properties, as described previously [44]. Additionally, silica (SiO_2_) possesses alkyl or polydimethylsiloxane chains, which contribute to hydrophobicity [45]. Furthermore, our findings supported our initial hypothesis that SiO_2_-deposited surfaces, which had WCA values of 118.1° ± 7.2°, had lower bacterial adhesion than plain PET samples (Figure 2E,F, Figure 4B–D and Figure 5B–D). These results were further supported by numerous studies showing lower bacterial adhesion on surfaces with higher WCA values [14,15,16,46,47]. Similar to our findings, Li et al. [47] demonstrated the fabrication of superhydrophobic silicone rubber (SR) surfaces using silica powder through a simple process and low-cost preparation based on the high-temperature vulcanized method. The results showed that the SiO_2_/SR sample still had excellent bouncing properties when the temperature was increased to 200 °C. The superhydrophobic properties of the SiO_2_-deposited surfaces were then quantified, revealing a WCA value of 160.3°. In addition, the antibacterial properties were also affected by the smooth surface topology of the film, resulting in the weakening of cell–surface interactions, regardless of the treatment, and thereby leading to unstable bacterial adhesion and detachment owing to mechanical stress, particularly during the rinsing process [15]. The anti-adhesive properties of SiO_2_-deposited surface was also confirmed by a roll-off angle test, indicating the non-sticky properties of the hydrophobic surface as water droplets were unable to spread and immediately slid off at 45° of inclination, whereas the plain PET surface showed that the water droplets were at pinning state as they could not easily roll off from the surface (Supplementary Information Video S1 and S2). A similar study by Schneider et al. [48] also displayed the high water repellency of silicon-deposited superhydrophobic surfaces on both static and dynamic contact angle tests, which can be explained by a high friction during the movement that increases driving force and surface tension of the liquid.

Furthermore, the association between surface topography and bacterial adhesion has also been reported as several studies have shown a reduction of bacterial growth when surface roughness is increased on the surface [31,32,33,34]. Similarly, our current study demonstrated that the deposition of SiO_2_ onto PET substrate increases the surface roughness (*R_RMS_* value of 82.50° ± 16.22° nm and *R_a_* value of 65.15 ± 15.26 nm, respectively), resulting in an increase in the WCA value to more than 90°, and both increased surface roughness and increased hydrophobicity of synergistically SiO_2_-PET substrate could reduce the bacterial adhesion on the surface (Figure 2E–G, Figure 4B–D and Figure 5B–D). This phenomenon is caused by the increased roughness on a hydrophobic surface that enhances air entrapment within the micro- or nano-structures, resulting in a lower penetration ability and free movement of the liquids; thus, the bacteria can be easily removed and detached from the surface [31]. Another similar study by Startek et al. [32] reported that the deposition of SiO_2_ with ten fluorinated carbons in the alkyl chain (-CF_10_) using spin-coating method onto PET substrate exhibits *R_RMS_* and *R_a_* values of 63.1 and 78.3 nm, respectively and increases the WCA value to 113°. In order to achieve superhydrophobicity, an increase of surface roughness is needed to reach a higher *R_RMS_* value, as reported by Ozkan et al. [33], who successfully fabricated a Cu-nanoparticles-coated polydimethylsiloxane (PDMS) substrate with WCA = 151° and *R_RMS_* = 230 nm. In their study, the superhydrophobic surface was able to weaken *E. coli* adhesion in just 15 min and *S. aureus* in 1 h. The relationship among surface roughness, hydrophobicity, and antibacterial properties was also shown by Svirinosky et al. [34], displaying an improvement of WCA value as surface roughness increases when different coating materials were incorporated onto the substrate that promotes the antibacterial functionality.

The antibacterial activity of silica coating was previously reported by Privett et al. [44]. In their study, silica colloids were applied for the fabrication of superhydrophobic surfaces, and the anti-adhesive ability was confirmed by the significant reduction of *S. aureus* and *P. aeruginosa* adhesion on the surface, with inhibition rates greater than 98% for both bacteria. In another similar study, Kaya et al. [49] successfully developed a drug carrier by utilizing silica hydrogels, and further investigation revealed its antibacterial activity against *E. coli*, *P. aeruginosa*, and *S. aureus*. Hence, our study is focused on the investigation of the antibacterial properties of silica-deposited PET that is fabricated by a novel electrospray technique. Based on the results, our silica fabricated surfaces were effective in inhibiting 66.30% *E. coli* and 64.09% *S. aureus* adhesion (Figure 4C and Figure 5C). These results were also supported by the fluorescence images that showed low numbers of attached bacteria on silica-deposited surfaces (Figure 4D and Figure 5D). Hasan et al. [50] previously reported that more than 85% of bacterial inhibition rate could be achieved by a surface with 80–300 nm of *R_a_* and 110–380 nm of *R_RMS_* values. As our fabricated surface displays both *R_a_* and *R_RMS_* values outside of these ranges, this might lower the antibacterial properties as it does not exhibit a WCA value greater than 150° (Figure 2E–G). Additionally, superhydrophobicity improves the bacterial inhibition rate to more than 90% as reported by numerous studies [12,34,45,51,52]. Although the antibacterial properties of electrosprayed SiO_2_ surfaces have not yet been extensively studied, several studies have demonstrated the effectiveness of electrospray coating at inhibiting bacterial adhesion using different materials, such as titania (TiO_2_) [16,17]. In general, surfaces with hydrophobic properties show reduced adhesion of bacteria compared with hydrophilic surfaces, which allow bacteria to bind [14]. A study conducted by Jalvo et al. [16] demonstrated that electrosprayed TiO_2_ coatings could inhibit biofilm formation by *S. aureus* in water filtration ceramic membranes. Irradiation treatment of TiO_2_-functionalized surfaces successfully inhibited *S. aureus* biofilm production with a 99% removal rate. Another similar study by Yoon et al. [17] also showed the antibacterial properties of electrosprayed TiO_2_-functionalized surfaces towards *E. coli*. The antibacterial test was conducted under ultraviolet radiation, and the effect was observed at an annealing temperature of 500 °C, showing a 93% reduction in *E. coli* growth.

Bacterial morphology is an essential factor that can affect bacterial adhesion during surface colonization. Indeed, in a previous study by Duvernoy et al., rod-shaped bacteria, such as *E. coli* and *Pseudomonas aeruginosa*, were shown to exhibit a strong adhesive force owing to their asymmetric microcolony shape [51]. Another approach by Hemmatian et al. [15] also showed a higher adherence of *E. coli* on hydrophobic surfaces compared with *S. aureus*, a circular-shaped bacterium, because rod-shaped bacteria have a larger interactive surface area. Furthermore, the cell wall structure and chemical composition are also involved in bacterial adhesion on the surface [52]. The bacterial cell wall is composed of peptidoglycan, which is structured around a poly-(*N*-acetylglucosamine-*N*-acetylmuramic acid) backbone [53]. Gram-positive bacteria differ from Gram-negative bacteria because of their thick peptidoglycan layer and the absence of lipopolysaccharide (LPS) [54]. Stronger inhibition of viable Gram-negative bacteria has also been reported in some studies [55,56]. For example, Lima et al. [55] revealed a higher antibacterial activity of chitosan-based hydrophobic surfaces against *P. aeruginosa* than *S. aureus*, which may be explained by the presence of phosphate groups in LPS from Gram-negative bacteria, enhancing the negative charge and promoting attraction to the positively charged chitosan surface. In contrast to previous reports, our findings revealed no significant differences in the inhibition of Gram-positive and Gram-negative bacterial adhesion, suggesting that the fabricated silica coating synthesized using our novel electrospray method may be effective for preventing the adhesion of both Gram-positive and Gram-negative bacteria. Moreover, bactericidal materials, such as nanomaterials, metal ions, and antimicrobial compounds, were not attributed to the fabricated surface, indicating that the biocompatibility and long-term stability were still well-maintained [57,58].

High biocompatibility of our fabricated surface was observed by more than 90% HDF cells under 24 h exposure of extracted coating materials (Figure 6A). These findings were consistent with the results of a previous study by Pillai et al. [59] who performed the same extraction method and showed that cell viability remained at greater than 80%; thus, these findings suggested that the coating materials did not diffuse out into the growth medium and exhibited excellent biocompatibility without toxicity in HDFs. In addition, our findings were similar to those of Dulski et al. who focused on the development of silver-silica coating as a functional biomaterial surface [60]. Based on their results, the fabricated surface could significantly inhibit *E. coli* and *S. aureus* biofilm formation and showed high biocompatibility with HDFs, yielding no dead or deformed cells after 72 h in morphological examinations. Owing to their biocompatibility, nanoparticles are often incorporated into SiO_2_ coatings to improve the mechanical stability of the material [61]. Navarro-Palomares et al. fabricated a surface coating by incorporating 15 and 30 µg/mL zinc oxide (ZnO) into silica and reported that the resulting product yielded over 75% viability in HeLa cells [62]. Thus, the SiO_2_ coating is not only biocompatible, but also exhibits biodegradable properties owing to its high stability under physical conditions. Nevertheless, some studies have also evaluated the potential cytotoxic effects of SiO_2_ nanoparticle coatings on human cells [63]. The results showed that the potential toxic effects do not originate from silica itself, but are a result of cooperation with co-nanoparticles, such as ZnO and TiO_2_, which could cause cell death at high concentrations by promoting the breakdown of the mitochondrial membrane [64]. Hence, our fabricated surface is a promising coating material for biomedical devices owing to its antibacterial properties and excellent biocompatibility.

## 4. Materials and Methods

### 4.1. Preparation of Deposition Materials

The deposition material used for surface fabrication was a mixture of silica and epoxy solutions. Fumed silica nanoparticles (Aerosil R 972) with a particle size of 16 nm were kindly donated by Evonik Industries (Essen, North Rhine-Westphalia, Germany), and epoxy resin (poly-[bisphenol A-co-epichlorohydrin]) and other reagents, such as 4,4-methylenebis [2-chloroaniline]) (a curing agent) and 2-butoxyethanol reagent, were purchased from Sigma-Aldrich (South Korea). To make the epoxy resin solution, 0.40 g epoxy resin (in pellet form), 0.08 g curing agent, and 9.52 g 2-butoxyethanol were mixed using a magnetic stirrer for 8 h at 70 °C. The silica solution was prepared by mixing 0.40 g fumed silica with 9.60 g 2-butoxyethanol using a magnetic stirrer for 2 h at room temperature. Both epoxy resin and silica solutions were mixed in a 1:1 ratio using a magnetic stirrer for 1 h at room temperature, followed by sonication for 5 min.

### 4.2. Electrospray-Based Silicon Deposition onto PET Films

A schematic illustration of the experimental setup is shown in Figure 1. Fabrication of the electrosprayed SiO_2_ surface was performed as reported in our previous study and we used this pre-optimized method in order to deposit SiO_2_ onto PET substrate with ~2 μm of thickness [23]. The deposition material was applied by 2 kPa air pressure to a syringe needle with a diameter of 150 μm. A 5 kV DC voltage was then applied from a power supply (SHV30R; Conver Tech, Gwangmyeong-si, Gyeonggi-do, Korea) to create an electric field and electrical charge in the liquid. To increase the uniformity of silica deposition, the PET film was placed at a distance of 5 cm from the nozzle tip, and an AC voltage of 3 kHz and 800 V was applied to the substrate holder. The AC signal was generated using an arbitrary function generator (Agilent 33220A; Agilent Technologies, Santa Clara, CA, USA), and the signal was then amplified using a high-voltage amplifier (Model 2220; Trek, Lockport, NY, USA). For better surface coverage, the distance between swaths was set to 0.5 cm, and the nozzle was moved in a horizontal grid motion with a scanning speed of 2 mm/s. The experiment was conducted at room temperature (25 °C). After the entire process was completed, the PET film was placed on a hot plate (SP131320-33; Thermo Scientific, Waltham, MA, USA) and annealed at 120 °C for 1 h.

### 4.3. Surface Characterization by FTIR, SEM, and AFM

The FTIR spectra of the epoxy resin, PET film, SiO_2_ powder, and SiO_2_-deposited PET film were analyzed by an Attenuated Total reflectance (ATR) technique using an FTIR spectrometer (Frontier; Perkin Elmer, Waltham, MA, USA) with a spectral width ranging from 450 to 4000 cm^−1^ at a spectral resolution of 1 cm^−1^, and the surface of the electrosprayed SiO_2_ deposition of PET films was examined using SEM (SIGMA 500; Carl Zeiss, Jena, Germany). Briefly, the specimens were gradually dehydrated using a graded series of ethanol solutions. After the specimens were completely dried, they were platinum coated using a sputter coater (E-1030; Hitachi, Tokyo, Japan) for 40 s prior to SEM imaging. The surface roughness of the PET film and SiO_2_-deposited PET film were evaluated by atomic force microscopy (AFM) (SPM 9700, Shimadzu, Japan) and root-mean-square roughness (*R_RMS_*) and average roughness (*R_a_*) were measured from the five random spots (scan area of 5 × 5 μm) by operating in a dynamic mode.

### 4.4. Contact Angle Measurement and Roll-Off Angle Test

The WCA values of the specimens were measured using the sessile drop method at 20 °C using a contact angle meter (KRUSS DSA30; Hamburg, Germany). A 5 µL droplet of distilled water was placed on the surface of the samples. All samples were evaluated at four and five different spots on SiO_2_-deposited PET and plain PET surfaces, respectively, and the results are presented as means ± standard deviations. To further confirm the anti-adhesive properties of the fabricated surfaces, roll-off angle test was performed by applying drop-by-drop water onto tilted-samples with an inclination angle of *δ* = 45° as previously described [65].

### 4.5. Bacterial Strains and Culture Conditions

Gram-positive bacteria (*S. aureus* KCTC3881) and Gram-negative bacteria (*E. coli* ATCC25922-GFP) were used to evaluate the antibacterial properties of the plain PET and SiO_2_-deposited PET samples. *S. aureus* and *E. coli* were purchased from the Korean Collection for Type Culture (KCTC, Daejeon, Korea) and American Type Culture Collection (ATCC, Manassas, VA, USA), respectively. Bacterial strains were preserved at −80 °C in LB broth, Miller (Difco Laboratories) containing 20% *v/v* glycerol (Bioshop GLY001.1). Before preparing the suspension culture, bacteria were subcultured on LB agar (LB broth Miller and Bacto Agar; Difco Laboratories) at 37 °C overnight. Single colonies from the streak plate were picked and inoculated into 5 mL LB broth in a round-bottom tube. The bacterial suspension was then incubated at 37 °C overnight in a shaking incubator at 200 rpm.

### 4.6. Assessment of Antibacterial Activity or Bacterial Adhesion

Antibacterial activity against *E. coli* and *S. aureus* was assessed, as illustrated in Figure 3. Bacterial suspensions were diluted serially to reach an OD_600_ value of 0.1, which is equivalent to a final concentration of 10^8^ CFU/mL (0.5 McFarland standard). One hundred microliters of each suspension were added and spread directly onto LB agar plates. The samples sized 1 × 1 cm were placed on top of the agar surface and incubated for 12 h at 37 °C. Samples were detached from the plate and rinsed gently in 1X PBS to remove non-adherent cells. Detached samples were placed in fresh LB broth and vortexed for 1 min. The liquid was transferred into a 48-well plate (500 µL per well), and absorbance was measured using an Epoch microplate spectrophotometer (Bio-Tek Instruments, Winooski, VT, USA) at 600 nm. The percentage of bacterial inhibition in the SiO_2_-deposited PET samples was calculated as follows:

(1) $$\mathrm{Inhibition}\;\left(\%\right)\;=\;\left(1-\frac{\mathrm{Average}\;\mathrm{absorbance}\;\mathrm{of}\;\mathrm{silica}\;\mathrm{deposited}\;\mathrm{PET}\;\mathrm{sample}}{\mathrm{Average}\;\mathrm{absorbance}\;\mathrm{of}\;\mathrm{plain}\;\mathrm{PET}\;\mathrm{sample}}\right)\times \;100\%$$

### 4.7. Live/Dead Assays for Bacteria

Live/dead assays were performed using a LIVE/DEAD BacLight Bacterial viability kit (cat# L7007, Thermo Scientific, Waltham, MA, USA) according to the manufacturer’s protocol. This assay was only performed for visualizing *S. aureus* because we used GFP-tagged *E. coli*. Samples were attached to the surface of the agar plate and detached after 12 h of incubation. Samples were then transferred to round-bottom tubes containing 1 mL LB broth and vortexed for 1 min to detach bacteria from the surface. The liquid was transferred into a 1.5 mL microcentrifuge tube and centrifuged at 10,000× *g* for 15 min. The staining solution was prepared by adding 3 µL dye mixture from the kit with a 1:1 ratio of components A and B in 500 µL LB broth. To visualize the presence of bacteria in the samples, 500 µL supernatant was discarded from the tube, and 503 µL of a previously prepared staining solution was added, followed by incubation for 15 min at room temperature in the dark. A 10 µL drop of the stained suspension was trapped between the glass slide and cover slip. Fluorescence imaging was performed using an SZX16 stereo zoom microscope (Olympus, Tokyo, Japan).

### 4.8. Cell Culture

HDFs (Daewoong Pharmaceutical Company, Seoul, Korea) were seeded into 96- and 24-well tissue culture plates at 5 × 10^4^ cells/cm, cultured in growth medium (DMEM (Corning, Oneonta, NY, USA) supplemented with 10% FBS (Gibco-BRL, Gaithersburg, MD, USA) and 1% P/S (10,000 U/mL penicillin and 10,000 g/mL streptomycin; Gibco-BRL)), and incubated at 37 °C in a humidified incubator with 5% CO_2_ for 24 h. Cells were then subjected to cytotoxicity tests when they reached 100% confluence.

### 4.9. In Vitro Cytotoxicity Tests and Cell Viability Analysis Using HDFs

Cytotoxicity tests were performed using MTT assays (Invitrogen Corporation, Carlsbad, CA, USA) in 96-well plates, as described previously [59]. Conditioned medium was prepared by incubating cells in growth medium for 24 h. The growth medium in 96-well plates was aspirated, and cells were washed with 1X PBS and incubated with conditioned medium for an additional 24 h. MTT assays were then performed by mixing 10 µL of 5 mg/mL MTT in 100 µL growth medium in each well, followed by a 2 h incubation. MTT solution was aspirated and DMSO (Samchun Chemicals) was added to each well, followed by gentle resuspension. The plates were incubated for 10 min, and the absorbance was measured using a microplate reader (Epoch Microplate Spectrophotometer; BioTek, Winooski, VT, USA) at 540 nm. Cell viability was calculated using the following formula:

(2) $$\mathrm{Viability}\;\left(\%\right)\;=\;\left(\frac{\mathrm{Average}\;\mathrm{absorbance}\;\mathrm{of}\;\mathrm{plain}\;\mathrm{PET}\;\mathrm{or}\;\mathrm{silica}\;\mathrm{deposited}\;\mathrm{PET}\;\mathrm{samples}}{\mathrm{Average}\;\mathrm{absorbance}\;\mathrm{of}\;\mathrm{cell}\;\mathrm{in}\;\mathrm{growth}\;\mathrm{media}}\right)\times \;100\%$$

To visualize cell morphology, cells were seeded at the same cell density as in MTT assays in 24-well tissue culture plates and cultured until they reached 100% confluence. The medium was then discarded and replaced with conditioned medium, and cells were incubated at 37 °C for an additional 24 h. For better visualization, cells were labeled with a fluorescence tracker (CellTracker Green CMFDA; Invitrogen Corporation). The staining dye was diluted in DMEM at a 1:1000 ratio and added to each well. Cells were then incubated at 37 °C for 40 min and washed with 1X PBS. Imaging was performed using a fluorescence microscope (Eclipse Ti-U; Nikon, Tokyo, Japan) at the Soonchunhyang Biomedical Research Core Facility of KBSI.

### 4.10. Statistical Analysis

All values are shown as means ± standard deviations of three replicates for each group, and statistical significance was assessed by one-way analysis of variance with Tukey’s multiple comparison tests using GraphPad Prism software. Results with *p* value less than 0.05 were considered significant (* *p* < 0.05; ** *p* < 0.01; *** *p* < 0.001 of three replicates).

## 5. Conclusions

In this study, we investigated the antibacterial properties of SiO_2_-deposited hydrophobic surfaces of PET films, which were developed using a novel electrospray-based method. Unlike the conventional electrospray technique, our approach features a novelty in the application of AC voltage under the substrate holder instead of ground (zero) voltage to enhance the efficiency and uniformity of silica deposition. This strategy successfully created a hydrophobic surface with antibacterial features against both Gram-positive and Gram-negative bacteria. The SiO_2_-deposited surface was characterized by SEM, FTIR, WCA, and AFM analyses. The hydrophobicity of the SiO_2_-deposited surface was confirmed (WCA value 118.1° ± 7.2°, *R_RMS_* value of 82.50 ± 16.22 nm, and *R_a_* value of 65.15 ± 15.26 nm), and the inhibition rates against *E. coli* and *S. aureus* were 66% and 64%, respectively. Furthermore, these results were consistent with the fluorescence images, which showed a low adherence of bacteria on the SiO_2_-deposited surface. We also performed cytotoxicity tests for in vitro cell culture and demonstrated greater than 90% viability in HDFs, suggesting that the SiO_2_-deposited surface had good biocompatibility. Therefore, our findings demonstrated that a novel electrospray based SiO_2_ deposition on to PET films could exhibit highly antibacterial and biocompatible properties and our fabricated hydrophobic surfaces may be suitable for use in various biomedical applications.

## Data Availability

The data presented in this study are available upon request from the corresponding authors.

## Supplementary Materials

The following are available online at https://www.mdpi.com/article/10.3390/ijms23010513/s1.

## Abbreviations

AC	Alternating current
AFM	Atomic force microscopy
CFU	colony forming unit
DC	direct current
DMEM	Dulbecco’s modified eagle’s medium
DMSO	dimethyl sulfoxide
EDTA	ethylenediaminetetraacetic acid
EPS	exopolysaccharide
FBS	fetal bovine serum
FTIR	Fourier-transform infrared spectroscopy
GFP	green fluorescent protein
HDF	human dermal fibroblast
LB	Luria-Bertani
LPS	lipopolysachharide
MTT	3-[4,5-dimethylthiazol-2-yl]-2,5 diphenyl tetrazolium bromide
OD	optical density
PBS	phosphate-buffered saline
PET	polyethylene terephthalate
PI	polyimide
P/S	penicillin-streptomycin
*Ra*	average roughness
*R_RMS_*	root-mean-square roughness
SEM	scanning electron microscope
SiO_2_	silicon dioxide
SR	silicone rubber
TiO_2_	titanium dioxide
WCA	water contact angle
ZnO	zinc oxide
