# Supplementary material for: A Novel Strategy for Creating an Antibacterial Surface Using a Highly Efficient Electrospray-Based Method for Silica Deposition
Source: Int J Mol Sci. 2022 Jan 3;23(1):513. doi: 10.3390/ijms23010513 (PMC8745460; doi:10.3390/ijms23010513)
Supplement: Supplementary file 1 [file ijms-23-00513-s001.zip › ijms-1523668 supplementary.pdf]

Supplementary Materials

A Novel Strategy for Creating an Antibacterial Surface Using a Highly Efficient Electrospray-Based Method for Silica Deposition

Odelia Levana [1,2,†], Soonkook Hong [3,†], Se Hyun Kim [4], Ji Hoon Jeong [1,2], Sung Sik Hur [1], Jin Woo Lee [5], Kye-Si Kwon [4,6,*] and Yongsung Hwang [1,2,*]

[1] Soonchunhyang Institute of Medi-Bio Science (SIMS), Soonchunhyang University, Cheonan-si 31151, Chungnam-do, Korea; odelialevana@sch.ac.kr (O.L.); jjh2020@sch.ac.kr (J.H.J.); sstahur@sch.ac.kr (S.S.H.)
[2] Department of Integrated Biomedical Science, Soonchunhyang University, Asan-si 31538, Chungnam-do, Korea
[3] Department of Mechanical and Naval Architectural Engineering, Republic of Korea Naval Academy, Changwon-si 51704, Kyungsangnam-do, Korea; hsk753@mnd.go.kr
[4] Department of Electronic Materials, Devices, and Equipment Engineering, Soonchunhyang University, Asan-si 31538, Chungnam-do, Korea; ksy9046@naver.com
[5] Department of Molecular Medicine, Gachon University College of Medicine, Incheon 21936, Korea; jwlee@gachon.ac.kr
[6] Department of Mechanical Engineering, Soonchunhyang University, Asan-si 31538, Chungnam-do, Korea
* Correspondence: kskwon@sch.ac.kr (K.-S.K.); yshwang0428@sch.ac.kr (Y.H.); Tel.: +82-41-530-1670 (K.-S.K.); +82-41-413-5017 (Y.H.)
† These authors contributed equally to this study.

This PDF file includes:

Supplementary Information Text
Supplementary Figure S1
Supplementary Figure S2
Supplementary Figure S3

Other Supplementary Materials for this manuscript include the following:

Video S1
Video S2

Supplementary Information Text

S1. Analysis of SiO_2-Deposited Layer Thickness on PET Substrate

We performed cross-sectional SEM images to assess the thickness of SiO_2-deposited layer on PET substrate. Samples were prepared by cutting the substrates using surgical blade and the surfaces of fractures were coated with Pt prior to the SEM analysis (Tescan SEM, Tescan Ltd., Brno, Czech Republic). Figure S1 showed that deposition of SiO_2 onto PET increased the substrate thickness to 1.71 μm.

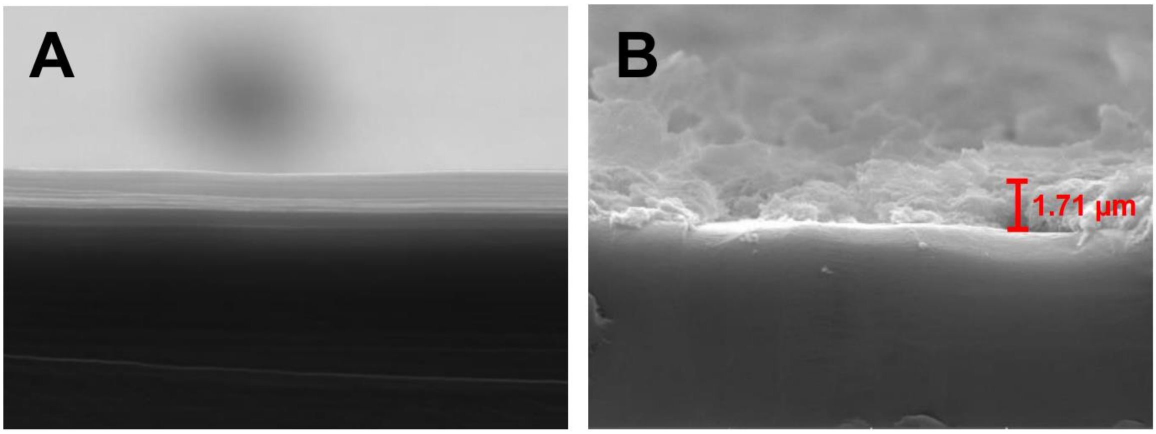

Figure S1. Cross-sectional SEM images of SiO_2-deposited layer. (**A**) A cross- sectional SEM image of plain PET film. (**B**) A cross-sectional SEM image of SiO_2- deposited PET film.

S2. FTIR Analysis

For better visualizing the vibrational fingerprint of SiO_2 and epoxy resin in FTIR spectra (refer to Figure 2D in the main text), the extended domain between 400 and 2000 cm^{-1} were presented in Figure S2.

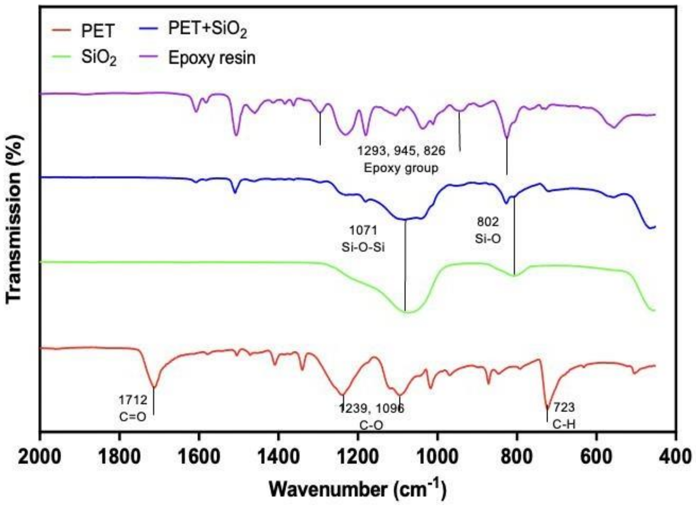

Figure S2. Bands responsible for SiO_2 and epoxy resin of plain PET and SiO_2- deposited PET samples in FTIR analysis.

S3. Water Contact Angle (WCA) Analysis

Figure S3 shows the water contact angle measurement on plain PET and SiO_2-deposited PET surfaces at 4 random spots. All results in the main text were presented as mean ± SD (refer to the Results and Discussions).

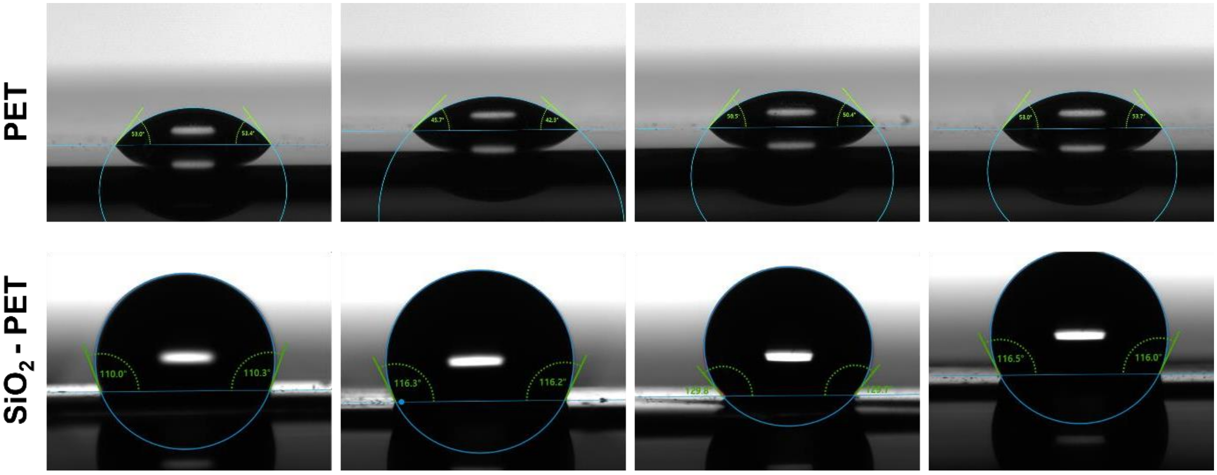

Figure S3. WCA analysis of plain PET and SiO_2-deposited PET samples.

S4. Dynamic contact angle analysis

Roll-off angle test was performed to observe the impact of wettability on water droplets dynamic of the plain PET and SiO_2-deposited samples (refer to the Materials and Methods in the main text). Results were shown in Video S1 and S2.

Videos:

Video S1: Roll-off angle test of plain PET film.
Video S2: Roll-of angle test of SiO_2-deposited film.
